# Metal accumulation in ecto- and endoparasites from the anadromous fish, the Pontic shad (*Alosa immaculata*)

**DOI:** 10.1017/S0031182021002080

**Published:** 2022-04

**Authors:** M. Nachev, D. Rozdina, D. N. Michler-Kozma, G. Raikova, B. Sures

**Affiliations:** 1 Department of Aquatic Ecology and Centre for Water and Environmental Research (ZWU), University of Duisburg-Essen, Essen, Germany; 2 Department of General and Applied Hydrobiology, Sofia University, Sofia, Bulgaria; 3 Institute for Landscape Ecology, University of Muenster, Muenster, Germany; 4 Department of Zoology, University of Johannesburg, Johannesburg, South Africa

**Keywords:** Bioindicator, Danube River, metals, monogeneans, nematodes

## Abstract

Among the parasitic taxa studied for their metal accumulation properties, especially Acanthocephala and Cestoda proved to be promising sentinels for metal pollution. However, studies on metal accumulation are still sparse for other parasite groups, mainly due to their small body size. In the present study, we collected the relatively large-sized monogenean *Mazocraes alosae* Hermann, 1782 from the gills of Pontic Shad (*Alosa immaculata* Bennet 1835) from its spawning region – the Danube River. The host tissues gills, muscle, intestine and liver, the monogeneans as well as the nematode *Hysterothylacium aduncum* (Rudolphi, 1802), in the cases of coinfected fish, were analysed for the elements As, Cd, Co, Cu, Fe, Mn, Pb, Se and Zn. All elements (except of As) were found in higher concentrations in monogeneans and nematodes compared to host muscle tissue. High bioconcentration factors were obtained for toxic elements such as Cd and Pb with concentrations being approximately 12 and 251 times higher in monogeneans and 773 and 33 in nematodes, respectively, as compared to host muscle tissue. In comparison to other host organs, however, some elements were found in similar or even lower concentrations in the parasites. Thus, monogeneans do not exhibit the high accumulation potential reported for other parasitic taxa. Physiological adaptations of the migratory host fish between freshwater and marine habitats with differences in uptake pathways and biological availability of elements can be discussed as a possible explanation for this divergent accumulation pattern.

## Graphical abstract

**Figure:**
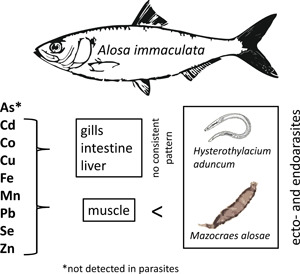

## Introduction

Different biological indicators can be applied for the assessment of metal pollution in aquatic habitats. Usually, these are free-living organisms, which are able to concentrate pollutants in their tissues and can simultaneously resist extended pollution levels (e.g. Phillips, 1977; Beeby, 2001; Gupta and Singh, 2011; Amoozadeh *et al*., 2014; Golestaninasab *et al*., 2014). Due to their high accumulation potential, pollutants occurring in very low concentrations in the environment can be detected by using such sentinel species, which would not be possible with conventional analyses of water, suspended particulate matter or sediments. Furthermore, bioindicators deliver valuable information about the biological availability of substances (Sures *et al*., 2017). Beside all well-established free-living biological indicators (e.g. different species of molluscs, crustaceans, annelids, fish), helminths of fish were also found to be very promising organisms to indicate metal pollution (summarized in Sures *et al*., 2017). Various species of acanthocephalans, cestodes as well as some parasitic nematodes were found to accumulate metals to extents, which exceeded those found in their host tissues (e.g. Sures *et al*., 1994*a*; Tenora *et al*., 2000; Baruš *et al*., 2007; Filipović Marijić *et al*., 2013, 2014; Brázová *et al*., 2015; reviewed also in Sures *et al*., 2017) and other free-living sentinels several folds (Sures *et al*., 1999*a*; Golestaninasab *et al*., 2014). However, until now mainly endoparasitic helminths were investigated with respect to their metal accumulation capacity. Information regarding monogeneans and other ectoparasites such as crustaceans is scarce or still missing (Pérez-del-Olmo *et al*., 2019). As ectoparasites are in direct contact with the surrounding environment, changes in aqueous metal levels may be rapidly reflected in accumulated metal levels in the parasites. Additionally, monogeneans can also take up metals from the mucous and tissues on which they feed.

The Pontic shad (*Alosa immaculata* Bennet 1835) belongs to the family Clupeidae. It is an anadromous migratory fish species, which typically inhabits the Black Sea and the Azov Sea. Since 2001 it was also reported in the Sea of Marmara (Eryilmaz, 2001). For spawning, the Pontic shad enters big rivers connected to the sea. It is an economically important fish species, whose annual catches in the Bulgarian sector of the Black Sea and the Danube River have dropped on average 3.5 times between 2003 and 2011 (Bulgarian Ministry of Agriculture and Food, personal communication). *A. immaculatа* was classified as a vulnerable species according to the IUCN red list of threatened species (IUSN, 2018) and the Bulgarian Red Book (Golemansky *et al*., 2015), included also in Annex 2 and 4 of the Bulgarian Biodiversity Act and Annex 2 of the Habitats Directive 92/43ЕЕС.

The parasite fauna of *A. immaculata* has been studied in different regions of the Ponto–Caspian area (Nizova and Syrovatka, 2000; Gaevskaya and Kornyychuk, 2003; Popjuk, 2009, 2011; Akmirza, 2013; Özer *et al*., 2013). The monogenean *Mazocraes alosae* Hermann, 1782 has been reported frequently from Pontic shad and other clupeids. This parasite exhibits a marine life cycle, however, it can resist the osmotic changes during the spawning migrations of it host (Aprahamian, 1985; Gérard *et al*., 2016). In contrast to many monogeneans of fish, *M. alosae* is characterized by large body size and therefore can provide sufficient sample material required for metal analyses. Accordingly, this species fulfils most of the basic criteria for parasites as bioindicators suggested by Sures (2003). Metal accumulation in tissues of Pontic shad (muscle, liver and gills) collected from the Danube River has been already studied by Visnjic-Jeftic *et al*. (2010). However, studies based on metal uptake in its helminth parasites are still missing. Changes in osmotic conditions and salinity, respectively, lead to various physiological adaptations in migratory fishes and influence the bioavailability of hydrophilic pollutants such as metals (Merian, 2004).

The aim of the study was to provide first data on the accumulation potential of monogeneans of fish. Therefore, a parasitological survey was carried out on Pontic Shad, which is known to harbour high infection rates of the relatively large-sized monogenean *M. alosae*. In order to evaluate its accumulation potential, the concentrations of different elements (As, Cd, Co, Cu, Fe, Mn, Pb, Se, Zn) in *M. alosae* were compared with those of host tissues (gills, muscle, intestine and liver) and with the nematode *Hysterothylacium aduncum* (Rudolphi, 1802), in case of co-infected fish hosts.

## Materials and methods

### Sample collection

Fish were collected by professional fishermen in April 2010 (*n* = 18) and 2011 (*n* = 22) during the spawning migration of the species. Sampling was performed along the Danube River in Bulgaria between the towns Lom and Vidin (river km 834–741) using gill nets with the size of the eye ranging between 32 and 88 mm. Prior to the parasitological investigation, each fish was measured for total length (TL) and standard length (SL) to the nearest mm and weighed with respect to the total weight (W) to the nearest g. The Fulton's condition factor (*K*) was calculated as follows *K* = 100 × W × TL^−3^ (see Nash *et al*., 2006). Parasitological analysis was performed using standard parasitological techniques with the aid of a stereomicroscope at a magnification ×40. The skin, fins, gills and internal organs as well as the body cavity were examined for parasite infestation. After dissection the prevalence and mean intensity of parasites were calculated according to Bush *et al*. (1997). Subsequently, the parasites, as well as samples of gills, liver, gonads, intestine, muscle and kidney, were collected and kept frozen at −20°C until metal analyses.

### Metal analyses

After defrosting, tissue samples and parasites were weighed and placed in 30 mL Teflon^®^ vessels (MarsXpress; CEM GmbH, Kamp-Lintfort Germany). Some of the parasite samples from different fish of the same year were pooled together in order to reach a wet weight of at least 10 mg. Subsequently, a mixture of 1.3 mL 65% nitric acid (HNO_3_ sub boiled) and 2.5 mL 30% hydrogen peroxide (H_2_O_2_ suprapure Merck, Germany) was added and vessels were heated up to 170°C for 40 min using a MARS 6 digestion system (CEM GmbH, Kamp-Linfort Germany). After digestion, the clear sample solution was brought to a volume of 5 mL using Mili-Q water.

Concentrations of nine elements (As, Cd, Co, Cu, Fe, Mn, Pb, Se, Zn) were measured using a quadrupole ICP-MS system (Elan 6000, Perkin Elmer, USA). Instrument calibration was performed using a dilution series (in range 0.1–100 ppb) from standard solutions (Merck, Germany, for details see Nachev *et al*., 2013). In order to prove the quality of our analytical procedure, fish tissue reference materials (IAEA-407, DORM3 and DOLT3) were analysed and accuracy rates were calculated.

### Data analyses and statistical treatment

To evaluate the metal accumulation capacity of fish helminths, bioconcentration factors were calculated according to Sures *et al*. (1999*b*), and concentration factors were calculated with respect to metal concentrations in the water. The necessary aqueous metal levels were obtained from the database of the International Commission for Protection of Danube River (ICPDR, 2016; see Table 1). The Mann–Whitney *U*-test was applied to compare the morphological data of fish collected in the years 2010 and 2011. For comparisons of metal levels in host tissues and parasites, the Wilcoxon matched-pair test was applied. Additionally, correlation analyses using Spearman rank correlations were performed in order to evaluate possible relationships between element concentrations of host tissues and parasites as well as the relationship between parasite fauna composition and fish morphology data.

**Table 1.** Concentrations (*μ*g/L) of selected elements in the river Daube at 834 km according to ICPDR (2016)

	2010	2011
As	1.8 (±0.3)	1.9 (±0.3)
Cd	n.d.	n.d.
Co	n.a.	n.a.
Cu	11.6 (±4.6)	8.0 (±8.3)
Fe	250.2 (±156.6)	222.7 (±309.6)
Mn	28.8 (±49.9)	13.8 (±5.5)
Pb	n.d.	n.d.
Se	n.a.	n.a.
Zn	10.3 (±5.1)	8.8 (±3.5)

n.d., values below the detection limit (provided as 0 in the database); n.a., no data available.

## Results

### Fish data

Morphological data on Pontic shad is given in Table 2. The fish from 2010 were significantly longer and heavier than those collected in 2011 (*P* < 0.001). However, the mean condition factor was similar for the fish from both years.

**Table 2.** Morphological data of Pontic shad

	2010 (*n* = 18)	2011 (*n* = 22)
TL (cm)	31.6 (±1.3)	23.0 (±3.4)
SL (cm)	27.2 (±1.2)	19.7 (±3.3)
H (cm)	5.9 (±0.4)	4.1 (± 0.7)
W (g)	226.0 (±37.0)	74.0 (±42.3)
*k*	0.9 (±0.2)	0.9 (±0.1)

TL, total length; SL, standard length; H, height; W, weight; k, condition factor.

### Parasitological data

Only the monogenean *M. alosae* and the nematode *H. aduncum* were recovered during the parasitological investigations. The nematodes were found in the stomach and intestine of the dissected fish, while the monogeneans were extracted from the gills. The prevalence and mean intensity of the monogeneans did not differ significantly between the fish collected in 2010 and 2011. However, there were clear differences in the prevalence of *H. aduncum* between the years (see Table 3) with the nematodes being more prevalent in the year 2010 than 2011. Significant correlations were detected between the presence of nematodes and fish weight (*r_s_* = 0.73), length (*r_s_* = 0.789) and height (*r_s_* = 0.64) considering data from both years, while the correlation between the nematodes and the condition factor of the fish was negative (*r_s_* = −0.649). There was no significant correlation between the monogeneans and fish morphological data and the parasites.

**Table 3.** Prevalence and intensity of infection of Pontic shad with the monogenean *Mazocraes alosae* and the nematode *Hysterothylacium aduncum*

Year	*P* (%)	MI (range)	MA
*M. alosae*			
2010	78	5.3 (1–16)	5.3
2011	68	4.3 (1–15)	2.9
*H. aduncum*			
2010	83	73.5 (1–350)	61.3
2011	5	16 (16)	0.7

P, prevalence; MI, mean intensity; MA, mean abundance.

### Element concentrations in the host and its parasites

**Fig. 1.**
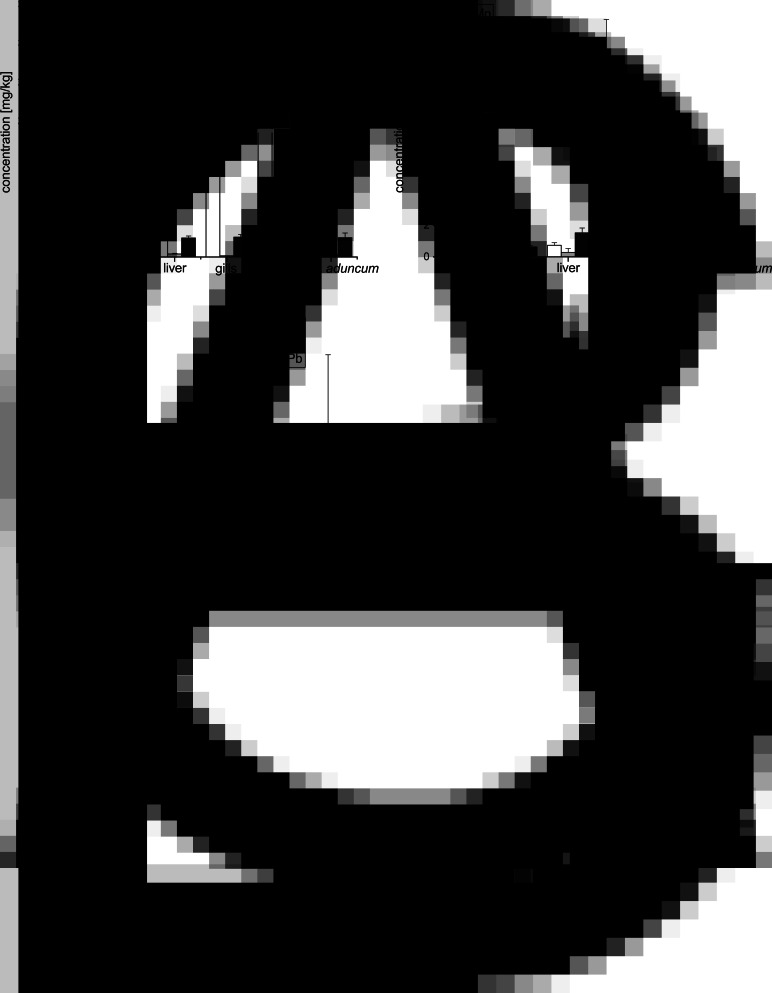
(A–C) Mean element concentrations (±s.d.) in the host–parasite system (*n* = 40). *Concentration below detection limit.

The certified elements of the reference materials showed recovery rates in the range of 100 ± 20% (see Table 4), which assured the required quality of the applied analytical procedure to quantify the elements. Comparison of element concentrations in the host–parasite systems revealed higher levels in the parasites than in host muscle (Fig. 1; Tables 5 and 6) with the exception of As, whose concentrations in both parasite species were below the detection limit. Interestingly, relatively high bioconcentration factors were obtained for Cd and Pb, with concentrations of Pb being approximately 251 and 33 times higher in the monogeneans and nematodes, respectively, compared to the host muscle. Cadmium showed a different pattern with a higher bioconcentration factor for nematodes (773) compared to monogeneans (12) with respect to host muscle. Both parasites also accumulated essential elements to a higher degree, particularly Co (nematodes) and Fe (monogeneans). The comparison of element concentrations between parasites and other fish organs showed less clear patterns. Generally, the order of element levels in host tissues was as follows: muscle < intestine < liver < gills (for details see Fig. 1 and Table 5). The element composition in gill tissue showed similar trends and concentrations as the monogeneans. With exception of Co, the element concentrations in nematodes were comparable to the tissues from their microhabitat (liver, intestine). Direct comparisons between both parasites revealed significantly higher concentrations for Fe, Pb, Se and Zn in monogeneans, whereas the nematodes accumulated Cd, Co and Mn to a significantly higher degree (see Fig. 1, Table 6). No significant temporal concentration differences between the years were observed in the host–parasite system, which corresponds with the aqueous element concentrations provided for the years 2010 and 2011 (see Table 1).

**Table 4.** Element concentrations in standard reference materials as well as accuracy and detection limits (DL) determined by ICP-MS analyses

	IAEA-407^a^			DORM 3^b^			DOLT 3^c^			DL
Element	Certified (mg kg^−1^)	Measured (mg kg^1^)	Accuracy (%)	Certified (mg kg^−1^)	Measured (mg kg^−1^)	Accuracy (%)	Certified (mg kg^−1^)	Measured (mg kg^−1^)	Accuracy (%)	(*μ*g L^−1^)
As	12.6	12.6	100%	6.88	6.53	95%	10.2	9.4	92%	0.014
Cd	0.18	0.17	94%	0.29	0.31	110%	19.4	20.8	107%	0.007
Co	0.1	0.1	112%	n.c.	–	–	n.c.	–	–	0.001
Cu	3.28	3.45	105%	15.5	15.5	100%	31.2	34.2	110%	0.047
Fe	146.00	143.26	98%	347	269	78%	1484	1225	83%	1.743
Mn	3.52	3.59	102%	n.c.	–	–	n.c.	–	–	0.001
Pb	0.12	0.13	109%	0.39	0.35	91%	0.32	0.27	85%	0.003
Se	2.83	3.29	116%	n.c.	–	–	7.06	6.47	92%	0.083
Zn	67.1	58.4	87%	51.3	44.1	86%	86.6	83.5	96%	0.078

a Fish homogenate.

b Dogfish muscle.

c Dogfish liver.

**Table 5.** Mean (±s.d.) bioconcentration factors for metal accumulation in parasites compared to different host tissues

Element	*M. alosae* C_(*M. alosae*)_/C_(organ)_ ± s.d.				*H.aduncum* C_(*H. aduncum*)_/C_(organ)_ ± s.d.				C_(*M. alosae*)_/C_(*H. aduncum*)_ ± s.d.
	Muscle	Gills	Intestine	Liver	Muscle	Gills	Intestine	Liver	
Cd	12.0 (±60.9)	0.7 (±0.4)	0.1 (±0.2)	0.1 (±0.1)	773.1 (±1483.3)	5.3 (±4.3)	0.6 (±0.9)	1.0 (±0.6)	0.2 (±0.1)
Co	6.2 (±4.2)	0.4 (±0.3)	2.9 (±2.5)	1.1 (±0. 8)	67.6 (±21.9)	4.2 (±1.8)	32.7 (±12.0)	12.0 (±5.1)	0.1 (±0.03)
Cu	6.0 (±5.5)	3.0 (±3.2)	2.6 (±2.3)	1.1 (±0.8)	3.9 (±2.1)	2.0 (±1.3)	1.5 (±0.7)	0.8 (±0.4)	1.3 (±1.2)
Fe	23.8 (±8.9)	1.3 (±0.5)	2.8 (±0.9)	1.0 (±0.5)	5.6 (±3.4)	0.3 (±0.2)	0.6 (±0.4)	0.3 (±0.1)	4.0 (±2.5)
Mn	4.7 (±4.6)	0.1 (±0.1)	3.8 (±3.6)	1.3 (±1.6)	6.3 (±1.9)	0.1 (±0.1)	4.4 (±1.9)	1.5 (±0.4)	0.6 (±0.5)
Pb	251.7 (±386.1)	0.8 (±0.7)	7.8 (±4.7)	11. 5 (±10.1)	33.5 (±55. 7)	0.2 (±0.2)	1.9 (±2.1)	2.8 (±3.1)	3.8 (±3.2)
Se	5.6 (±4.1)	0.4 (±0.2)	3.2 (±2. 7)	1.2 (±0.9)	1.9 (±1.1)	0.2 (±0.1)	0.8 (±0.3)	0.4 (±0.2)	4.8 (±7.2)
Zn	11.9 (±3.4)	1.6 (±0.5)	3.0 (±1.0)	1.6 (±0.4)	7.8 (±1.7)	1.0 (±0.2)	1.8 (±0.5)	1.0 (±0.2)	1.7 (±0.8)

**Table 6.** Differences between element concentrations in fish organs and parasites, and between *M. alosae* and *H. aduncuum*

	*M. alosae*				*H.aduncum*				*M. alosae vs H. aduncum*
Element	M.a. ↔ M	M.a. ↔ I	M.a. ↔ L	M.a. ↔ G	H.a. ↔ M	H.a. ↔ I	H.a. ↔ L	H.a. ↔ G	M.a. ↔ H.a.
As	M^a^	I^a^	L^a^	G^a^	M^a^	I^a^	L^a^	G^a^	n.s.^a^
Cd	M.a.**	I**	L**	n.s.	H.a.**	I*	n.s.	H.a.**	H.a.**
Co	M.a.**	M.a.**	n.s.	G**	H.a.**	H.a.**	H.a.**	H.a.**	H.a.**
Cu	M.a.**	M.a.*	n.s.	M.a.**	H.a.**	n.s.	n.s.	H.a.*	n.s.
Fe	M.a.**	M.a.**	n.s.	n.s.	H.a.**	I*	L**	G**	M.a.**
Mn	M.a.**	M.a.**	n.s.	G**	H.a.**	H.a.**	H.a.**	G**	H.a.*
Pb	M.a.**	M.a.**	M.a.**	n.s.	H.a.**	n.s.	H.a.*	G**	M.a.**
Se	M.a.**	M.a.**	n.s.	G**	H.a.**	n.s.	L**	G**	M.a.*
Zn	M.a.**	M.a.**	M.a.**	M.a.**	H.a.**	H.a.**	n.s.	n.s.	M.a.**

M, muscle; I, intestine; L, liver; G, gills; M.a., *M. alosae*; H.a., *H.aduncum*; n.s., not significantly different.

a Not statistically tested as concentrations in parasites were below the detection limit.

In case of significant difference, the site for higher concentration is given in each cell.

*Significant at *P* ⩽ 0.05.

**Significant at *P* ⩽ 0.01.

## Discussion

In this paper, we studied the metal accumulation potential of the monogenean *M. alosae* and compared it with that of the nematode *H. aduncum* from the same fish host and different tissues of the host. In general, both parasite species had higher levels of all elements than muscle tissue of the host with the exception of As, whose concentrations were below the detection limit in the parasite samples. The non-essential elements Cd and Pb were found in much higher levels in the parasites than in the host muscle tissues similar to already published data for other fish helminths such as acanthocephalans, cestodes and nematodes (Sures *et al*., 1994*a*, 1994*b*; Tenora *et al*., 2000; Schludermann *et al*., 2003; Baruš *et al*., 2007; Nachev *et al*., 2013; Brázová *et al*., 2015). Currently, there is not much information available on monogeneans. A study on the fresh-water monogenean *Ancyrocephalus mogurndae* (Yamaguti, 1940) provided evidence that it can also accumulate toxic elements such as Pb to a higher extent than its host tissues (Qian and Pin, 2000). However, the authors did not provide the exact levels of Pb in the host–parasite system and did not calculate bioconcentration factors of monogeneans with respect to host tissues or to water. Regarding systematically related taxa such as digeneans, Sures *et al*. (1998) reported concentrations of Pb in the digenean *Fasciola hepatica* Linnaeus, 1758 from cattle, which were up to 170 times higher than its host tissues. In the study of Lotfy *et al*. (2013) Pb concentrations in *F. hepatica* and *F. gigantica* Cobbold, 1855 were about 2.5 times higher than in host liver tissue, in contrast to Cd that showed comparable or lower concentrations in the parasites compared to host tissues. Tellez and Merchant (2015) also provided evidence that other digeneans can accumulate various essential and non-essential elements at concentrations more than 1000 times higher than their alligator host tissues. However, almost all data on trematodes refer to terrestrial host–parasite associations (see Sures *et al*. (2017) and there are only a few studies, which focused on marine (Torres *et al*., 2014) or limnetic ones (Akinsanya and Kuton, 2016).

Previous studies on the accumulation potential of some species of the genus *Hysterothylacium* demonstrated the ability of this nematode to accumulate different metals. Among the elements, Cd and Pb were almost always reported at higher concentrations in the parasite than in the host (Dural *et al*., 2010, 2011; Abdel-Ghaffar *et al*., 2014; Mazhar *et al*., 2014), with occasional reports of more than 1000 times higher concentrations in the nematode (Dural *et al*., 2010). In our study, *H. aduncum* was also found to show high accumulation rates for Cd with respect to the host muscle tissue (see Table 5). However, Pb showed an opposite pattern being much higher accumulated in tissues of *M. aloesae* than in nematodes.

Generally, the biological availability of metal ions is much higher in limnetic than in marine habitats, as the concentration of hydrated ions increases with decreasing salinity (Merian, 2004). As anadromous fish, *A. imaculata* experiences physiological adaptations during its migration activities. Changes in the salinity trigger adaptations in the host (Kültz, 2011), which on the other hand alter the uptake mechanisms/routes of metals by the fish (Merian, 2004). Thus, the uptake of metals in the marine environment occurs mainly over the ingested water and food. In freshwater, the uptake largely occurs through the gills, as the osmotic differences between body fluids of fish and freshwater cause water and dissolved elements to pass through the gill membrane. Thereafter, metal ions enter the blood system of fish where they might become available for blood-sucking parasites. With the aid of metal excretion into the intestine and the subsequent enterohepatic circulation, metal ions can also be taken up by endohelminths (Sures and Siddall, 1999).

Ectoparasites and monogeneans, in particular, can take up metals most probably *via* two routes. The first one represents the direct uptake from the water through the body surface similar to other platyheminthes dwelling in the intestinal lumen. The second one represents the dietary uptake. *M. alosae* is a haematophagous ectoparasite (Gérard *et al*., 2016) and therefore it can accumulate metals available in the circulation system of the host. This was evident from the relatively high iron levels obtained for the monogeneans in comparison to the host's tissues, as haemoglobin in blood contains iron (Merian, 2004). Thus, the accumulation of metals in monogeneans is mediated by the biological availability of metals in the water and/or by the physiology of the host. Although the concentrations of Cd and Pb in the water were below the limits of detection (see Table 1), the monogeneans accumulated up to 12 times more Cd and up to 250 times more Pb than the host's muscle. However, the concentrations of both elements were in a similar range with those in the gills tissues assuming that non-essential elements in the monogeneans reach equilibrium with respect to the tissue of the parasites microhabitat. Accordingly, the uptake routes and their importance for metal accumulation in ectoparasites cannot be clearly described.

An almost similar trend was observed for the element Pb in *H. adunculum* with respect to its microhabitat (intestine). The patterns for Cd were not that clear due to the high deviation of the concentrations in the intestine. As previously described, the most important accumulation pathway for fish and gut parasites in freshwater habitats represents the enterohepatic circulation (Nachev and Sures, 2016). Thus, the metal accumulation process is constrained mainly by the physiology of the host with osmotic adaptations during the spawning migration being a significant factor.

Here, we provide the first detailed information about the element accumulation of a monogenean species infecting a migratory (anadromous) fish. In general, the monogeneans are expected to resemble the accumulation capacity of homologues taxa e.g. from the phylum Plathyhelminthes. However, *M. alosae* does not exhibit such a high accumulation potential, with some elements being found in higher concentrations even in host tissues. This could be a result from the migratory behaviour of the fish host and the fact that *M. alosae* as an ectoparasite needs to adapt to its physiological adaptations and to osmotic changes. *M. alosae* can survive during the migration, however, the differences between freshwater and marine habitats and in particular the differences in the biological availability of metals, the uptake and elimination rates as well as different pollution patterns along the Danube River can shape the accumulation patterns within ectoparasites. Therefore, surveys in marine habitats can provide more representative information about the pollution levels, as the host–parasite system is exposed for a longer period to similar conditions in comparison to the short time during the spawning migration. In general, species with similar traits as *M. alosae* could be the subject of future research aiming to study the sentinel properties of monogeneans. *M. alosae* is highly abundant in different clupeid species (Gérard *et al*., 2016) and with its large body size, it fulfils most of the important criteria for bioindicators as suggested by Sures (2003). Moreover, during the survey in 2010 and 2011 the prevalence and the intensity of monogeneans did not change in contrast to the nematodes that have been less prevalent in the fish collected in 2011. Latter differences could be attributed to the size of the fish and a different diet of larger and smaller individuals, respectively. In contrast to smaller *A. immaculata* specimens, the larger ones become piscivorous and feed on small fish, which serve as the second intermediate or paratenic host for *H. aduncum* and harbour/accumulate the larval nematodes, respectively (Hoestlandt, 1991; Koie, 1993). Accordingly, there was a positive correlation between infrapopulation size of *H. aduncum* with fish size and weight that help to explain much higher infection levels in the larger fish individuals collected in 2010 (see Tables 2 and 3). In contrast, host size and diet played no significant role in the distribution and abundance of monoxenous parasites and monogeneans in particular. Although the infestation with a high number of blood-feeding monogeneans anchored in the gill tissue can result in severe anaemia of the fish host (Buchmann and Bresciani, 2006; Gérard *et al*., 2016) our study did not exhibit any obvious effect of *M. alosae* on the health status (i.e. condition factor) of the fish. There was no significant relationship between infection levels and fish condition factors, as previously reported by Gérard *et al*. (2016) for *M. alosae* on *A. alosa* and *Alosa fallax*.
